# Exploration of the clinical benefits of sodium glucose co-transporter 2 inhibitors in diabetic patients with concomitant heart failure

**DOI:** 10.1186/s12933-018-0719-7

**Published:** 2018-05-25

**Authors:** Atsushi Tanaka, Koichi Node

**Affiliations:** Department of Cardiovascular Medicine, Saga University, 5-1-1 Nabeshima, Saga, 849-8501 Japan

## Abstract

Prevention and treatment strategies for heart failure (HF) in diabetes have not been fully established, at least partly due to lack of recognition of a pathological link between the two and effective antidiabetic agents for HF. Recent cardiovascular (CV) outcomes trials demonstrated that treatment with sodium glucose co-transporter 2 (SGLT2) inhibitors greatly improved major CV adverse events in type 2 diabetes (T2D) patients at high risk for CV events, seemingly driven by risk reduction in HF-related outcomes. The beneficial effects of SGLT2 inhibitors on such outcomes and the heart itself are unique characteristics among antidiabetic agents, and SGLT2 inhibitors are expected to be a promising therapeutic option for CV disease and HF care. However, because a limited number of T2D patients with concomitant HF were included in the CV outcomes trials, the treatment effects of SGLT2 inhibitors for such conditions have not been fully investigated. Moreover, there has been little evidence to suggest SGLT2 inhibitor mediated effects on CV function and relevant biomarkers. Januzzi et al. (J Am Coll Cardiol 70: 704–712, 2017) reported that canagliflozin treatment could delay the escalation of cardiac biomarkers in older T2D patients, suggesting direct CV protection by SGLT2 inhibitors in this population. Whether SGLT2 inhibitors can exert similar benefits in T2D patients with concomitant HF will likely be the next big issue of medical concern. Furthermore, newer clinical trials are currently ongoing to investigate whether SGLT2 inhibitors exhibit beneficial effects for HF, both in the presence and absence of T2D. Such trials may potentially identify novel approaches for treating HF.

Januzzi et al. [1] reported that treatment with canagliflozin, a sodium glucose co-transporter 2 (SGLT2) inhibitor, attenuated serial escalation of cardiovascular (CV) biomarkers, including N-terminal pro-B type natriuretic peptide and high-sensitivity troponin I, over a 104-week period compared with placebo, in older adults with type 2 diabetes (T2D). Measurement of CV biomarkers has an established role in the diagnosis of CV disease and prediction of prognosis in clinical settings, including diabetes care, due to the specificity to the CV system [2]. Therefore, elucidation of how such biomarkers are influenced by medical intervention is worthy of remark.

Accumulating evidence suggests that SGLT2 inhibitors provide multiple benefits beyond glucose-lowering, for both CV and renal regulation, leading to improved CV outcomes. The EMPA-REG OUTCOME trial [3] and CANVAS Program [4] demonstrated that SGLT2 inhibitor treatment significantly reduced major CV adverse events and hospitalization for heart failure (HF) in patients with T2D at high risk of CV events. However, as the exact mechanisms by which SGLT2 inhibitors exert their beneficial effects were minimally investigated in these trials, the effect of treatment on the whole CV system is poorly understood. Meanwhile, CV benefits, especially reduced HF hospitalization, were noted during the early-phase of SGLT2 inhibitor treatment and continued over time in the CV outcomes trials. Interestingly, these effects were also consistent with attenuation of serial escalations of CV biomarkers with canagliflozin treatment [1]. The authors noted that although canagliflozin-mediated effects on CV biomarkers may be associated with the CV benefits observed in the recent CV outcomes trials, further studies are needed to assess any direct and longitudinal links between changes in biomarkers and CV outcomes. In addition, other functional tests (e.g., echocardiography) should help elucidate what the biomarker changes reflect mechanistically. A previous preliminary study successfully demonstrated that short-term treatment with empagliflozin was associated with promotion of left ventricular reverse remodeling and improved index of diastolic function in patients with T2D and established CV disease [5].

Importantly, patients with a history of established CV disease and advanced HF were excluded from the study [1]. In fact, baseline biomarker levels and the range of changes were modest. This begs the question: what are the effects of SGLT2 inhibitors on CV biomarkers in high-risk patients? In particular, whether SGLT2 inhibitors could be a favored therapeutic tool for HF itself seems to come under the spotlight [6]. However, only a limited number (approximately 10–14% of all participants) of T2D patients with a history of HF were included in the CV outcomes trials [3, 4]. Furthermore, in the sub-group analyses stratified by the presence or absence of HF at baseline, the treatment effects of SGLT2 inhibitors on CV death and hospitalization for HF in each subgroup were inconsistent between the trials (Fig. 1) [7, 8]. Thus, CV safety and efficacy of SGLT2 inhibitors in T2D patients with concomitant HF remain to be fully investigated. Therefore, ongoing prospective trials examining the effects of SGLT2 inhibitors on CV biomarkers in T2D patients with documented HF may further elucidate the underlying mechanisms and clinical application of SGLT2 inhibitors in CV disease and HF care [9].

**Fig. 1:**
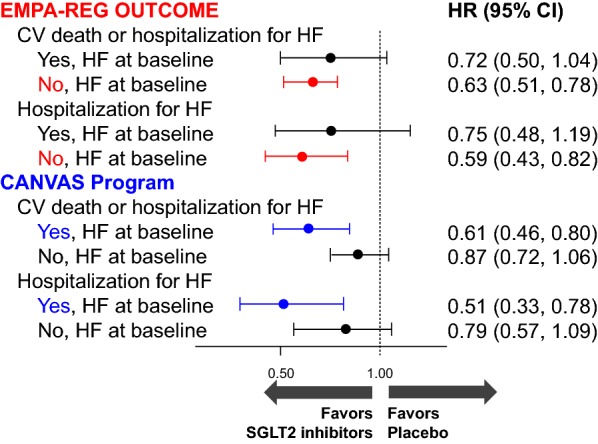
Comparison of the treatment effects of SGLT2 inhibitors on CV death and hospitalization for HF, stratified according to the presence or absence of history of HF at baseline. In the EMPA-REG OUTCOME trial, both outcomes were significantly decreased in the subgroups without HF at baseline, while in the CANVAS Program, the outcomes were reduced in the subgroups with HF at baseline, although there was no statistically significant treatment effect across the subgroups. *CV* cardiovascular, *HF* heart failure, *SGLT*2 sodium glucose co-transporter 2

## Abbreviations

CV: cardiovascular
HF: heart failure
SGLT2: sodium glucose co-transporter 2
T2D: type 2 diabetes
